# Laminin-511-E8 promotes efficient in vitro expansion of human limbal melanocytes

**DOI:** 10.1038/s41598-020-68120-0

**Published:** 2020-07-06

**Authors:** Naresh Polisetti, Andreas Gießl, Shen Li, Lydia Sorokin, Friedrich E. Kruse, Ursula Schlötzer-Schrehardt

**Affiliations:** 1 Department of Ophthalmology, University Hospital Erlangen, Friedrich-Alexander-University of Erlangen-Nürnberg, Schwabachanlage 6, 91054 Erlangen, Germany; 2 Eye Center, Medical Center - Faculty of Medicine, University of Freiburg, Freiburg, Germany; 3 Institute of Physiological Chemistry and Pathobiochemistry and Cells-in-Motion Interfaculty Centre (CIMIC), University of Münster, Münster, Germany

**Keywords:** Medical research, Eye diseases

## Abstract

Limbal melanocytes, located in the basal epithelial layer of the corneoscleral limbus, represent essential components of the corneal epithelial stem cell niche, but, due to difficulties in their isolation and cultivation, their biological roles and potential for stem cell-based tissue engineering approaches have not been comprehensively studied. Here, we established a protocol for the efficient isolation and cultivation of pure populations of human limbal melanocytes, which could be expanded at high yield by using recombinant laminin (LN)-511-E8 as culture substrate. Co-cultivation of limbal melanocytes with limbal epithelial stem/progenitor cells on fibrin hydrogels pre-incubated with LN-511-E8 resulted in multilayered stratified epithelial constructs within ten days. By reproducing physiological cell–cell and cell–matrix interactions of the native niche environment, these biomimetic co-culture systems provide a promising experimental model for investigating the functional roles of melanocytes in the limbal stem cell niche and their suitability for developing advanced epithelial grafts for ocular surface surface reconstruction.

## Introduction

The limbal stem cell niche refers to a specific anatomic location of the ocular surface which harbors a population of stem cells that regulate homeostasis and repair of the corneal epithelium^1^. Its specialized structure allows direct or indirect interactions of limbal epithelial stem and progenitor cells (LEPCs) with non‐epithelial supporting niche cells, blood vessels, and nerves across a specialized basement membrane^2^. Limbal melanocytes (LMs), located in the basal epithelial layer of the corneoscleral limbus, have been long known as essential components of the limbal stem cell niche^3–6^. They are each associated with about 10 LEPCs forming clusters resembling the melanin units in the skin^4^. Their main function is considered the transfer of melanin granules to neighboring LEPCs to protect them from UV damage. Accordingly, the degree of pigmentation was found to correlate with the state of LEPC differentiation, with the most highly pigmented populations being the most immature of the progenitor cells^7^. A prerequisite for this task is the close contact between LMs and LEPCs, which has been reported to be mainly mediated by E- and P-cadherins^8^. In addition to their major function of photoprotection, LMs isolated from limbal biopsies have been also shown to support the growth and maintenance of LEPCs in tissue engineered corneal epithelial constructs^9,10^. These observations suggest that LMs may have additional biological functions in the limbal stem cell niche, and that co-cultivation of both LEPCs and LMs could represent an improved novel strategy for cultivated limbal epithelial transplantation, which has been evolved as the first line therapy for patients suffering from limbal stem cell deficiency^11^.

Whereas limbal mesenchymal stromal cells (LMSCs), which represent another supporting cell type of the limbal niche^8^, have been widely studied for their beneficial effects on LEPC function and cultivation^12–14^, little attention has been paid to the role of LMs in LEPC function and LEPC-based stem cell therapies. This deficit may be attributed to low LM density in the niche zone and their low proliferative activity, complicating their isolation and cultivation. In order to obtain sufficient cell numbers of pure human LMs for tissue engineering approaches or functional analyses, respectively, we aimed at developing a protocol for the efficient isolation and cultivation of LMs using appropriate culture substrates specifically expressed in the limbal niche.

Previous studies showed that laminin (LN)-332 and LN-511 promoted adhesion, migration and differentiation of epidermal melanocytes^15–17^. We have recently reported that the LN chains α2, α3, α5, β1, β2, β3, γ1, γ2 and γ3 are strongly expressed in the limbal basement membrane, and that the α5-containing isoforms LN-511 and LN-521 enabled efficient expansion of LEPC in both 2D and 3D cultures^18^. LN-511-E8, representing the integrin-binding biologically active C-terminal portion of LN-511, also supported the efficient expansion of LEPC similar to the full-length isoform. Since LMs reside at the epithelial basement membrane in close spatial association with LEPCs, we hypothesized that LN-511-E8 may also promote ex vivo expansion and maintenance of this niche cell population. In this study, we provide guidelines for the efficient isolation and cultivation of primary human LMs. Using recombinant LN-511-E8 fragments, we obtained high yields of pure LMs, which could be successfully applied for tissue engineering of fibrin-based corneal epithelial constructs. Such biomimetic 3D co-culture systems of LMs and LEPCs may represent powerful tools for studying stem cell-niche cell interactions and for ocular surface reconstruction.

## Results

### Generation of purified limbal melanocyte cultures

**Figure 1:**
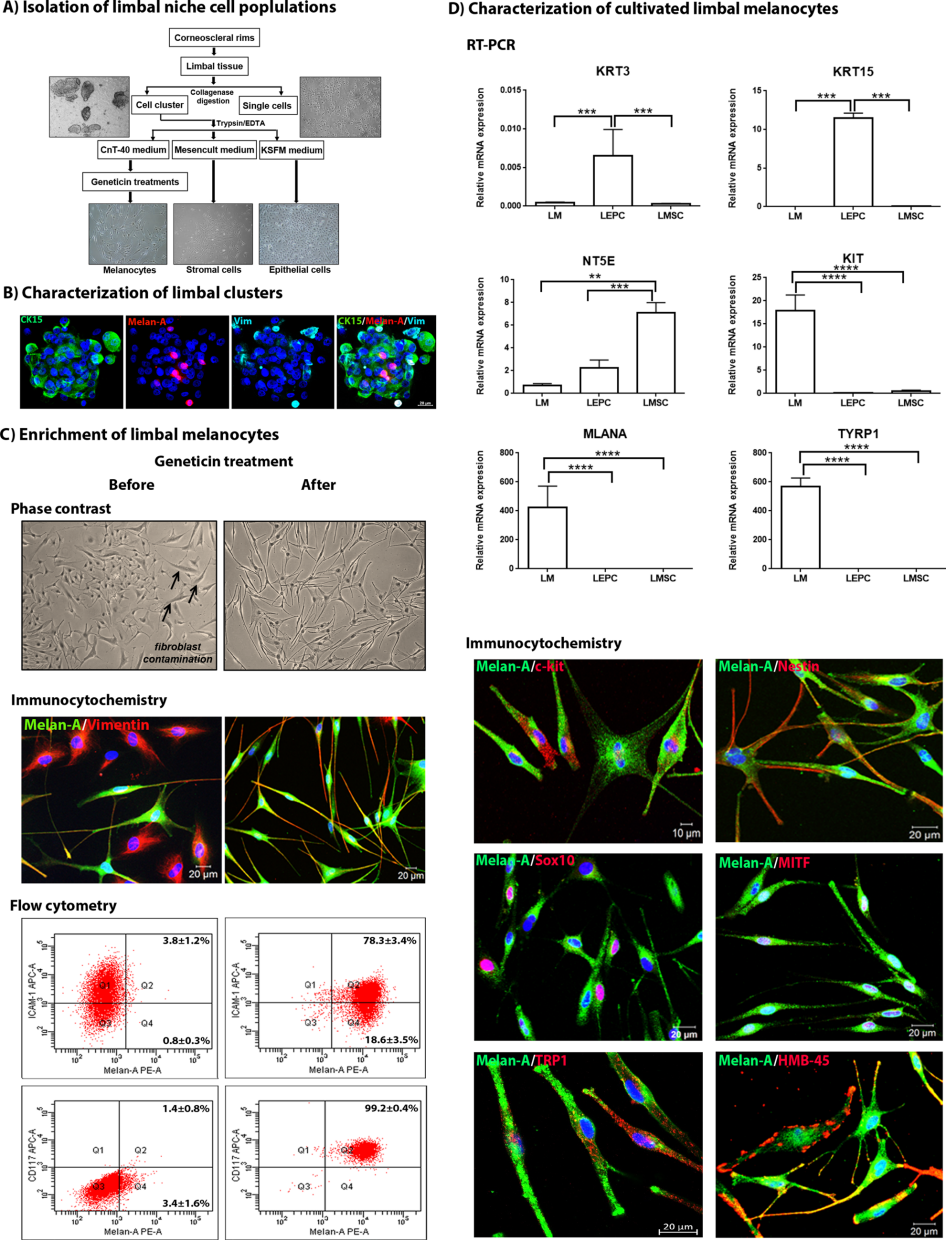
Isolation, cultivation and characterization of limbal melanocytes. (**A**) Graphical representation of limbal cell isolation: Collagenase digestion of limbal tissue generated single cells and cell clusters, which were dissociated into single cells by trypsin/EDTA and cultured in specific media to enrich epithelial cells, mesenchymal stromal cells and melanocytes. (**B**) Immunofluorescence triple staining of limbal cell clusters showing cells positive for cytokeratin (CK) 15 (green), Melan-A (red) and vimentin (Vim) (turquoise); nuclei are counterstained with 4′,6‐diamidino‐2‐phenylindole (DAPI, blue). Scale bar: 20 µm. (**C**) Enrichment of limbal melanocytes before (left column) and after geneticin treatment (right column): Phase contrast images (top) showing contamination with stromal fibroblasts (arrows) and purified melanocytes, respectively. Double immunolabeling (center) showing presence of Melan-A (green)^−^/vimentin (red)^+^ cells before and pure populations of Melan-A^+^/vimentin^+^ cells after geneticin treatment; nuclear counterstaining with DAPI (blue). Flow cytometry analysis (bottom) showing an increase of Melan-A^+^/ICAM-1^+^ cells and almost pure populations of Melan-A^+^/CD117^+^ cells after geneticin treatment. Data (% of positive cells) are expressed as means ± SEM (n = 3). (**D**) Quantitative real-time polymerase chain reaction (qRT-PCR) primer assays confirming differential expression of established epithelial (KRT3, KRT15), mesenchymal (NT5E), and melanocyte (KIT, MLANA, TYRP1) markers in cultured LEPC, LMSC, and LMs, respectively. Data are normalized to GAPDH and expressed as means (2^−ΔCT^ × 1,000) ± SEM (n = 5). *p < 0.05; **p < 0.01; ***p < 0.001; Mann–Whitney *U* test. Immunofluorescence double labeling of Melan-A (green) with c-Kit, nestin, Sox-10, MITF, TRP1, and HMB-45 (red); nuclear counterstaining with DAPI (blue). (CK15, cytokeratin 15; ICAM-1, intercellular cell adhesion molecule 1; LEPC, limbal epithelial progenitor cells; LMSC, limbal mesenchymal stromal cells; LM, limbal melanocytes; KRT, keratin; NT5E, 5′-ecto nucleotidase; Sox10, sex related HMG box 10; TYRP1/TRP1, tyrosinase related protein 1; HMB-45, human melanoma black-45; MITF, micropthalmia associated transcription factor).

Limbal niche cell populations, comprising LEPCs, LMSCs and LMs, were isolated from limbal tissue specimens and differentially enriched by using specific culture media and sequential purification steps as summarized in Fig. 1A. Collagenase A digestion of limbal tissue specimens generated both single cells and cell clusters, which contained all niche cell populations and stained positive for epithelial progenitor cell marker (cytokeratin 15), mesenchymal stromal cell marker (vimentin) and melanocyte marker (Melan-A) (Fig. 1B). Individual cells were released from the cell clusters by trypsin/EDTA treatment and were seeded into three separate culture flasks containing cell-type specific culture media (Fig. 1A). After 48 h of plating, small clusters of melanocyte-like dendritic cells were observed, together with contaminating epithelial and fibroblast-like cells, in the CnT-40 containing flasks.

A low concentration of trypsin (0.05%) was used to enzymatically separate epithelial cells from fibroblast-like and melanocyte-like cells. The remaining cell cultures still contained a large proportion of contaminating fibroblasts, which were vimentin^+^/Melan-A^−^ by immunocytochemistry and ICAM-1^+^/Melan-A^−^/CD117^−^ by flow cytometry (Fig. 1C, left column). After 3 cycles of treatment with geneticin, an inhibitor of protein synthesis, relatively pure cultures of Melan-A^+^/vimentin^+^ melanocytes were obtained (Fig. 1C, right column). Flow cytometry showed that the small fraction of Melan-A^+^/ICAM-1^+^ cells increased from 3.8 to 78.3%, indicating that melanocytes partially express ICAM-1^19^, and that Melan-A^+^/CD117^+^ cells increased from 1.4 to 99.2%, indicating an almost 100% pure melanocyte population after geneticin treatment (Fig. 1C, right column)^20^.

To verify the purity of LM cultures, expression profiles of known positive and negative melanocyte markers were analyzed on the mRNA and protein level in comparison with cultivated LEPCs and LMSCs. qPCR showed high expression levels of typical melanocyte markers, including CD117/c-Kit (KIT), Melan-A (MLANA), and tyrosine-related protein (TYRP1)^20,21^, whereas corneal epithelial markers, such as cytokeratin 3 (KRT3) and cytokeratin 15 (KRT15), and mesenchymal stem cell markers, such as CD73 (NT5E), were not expressed in the enriched LM populations (Fig. 1D). Doubling labeling immunocytochemistry showed colocalization of Melan-A with c-Kit, nestin, SRY-box transcription factor 10 (Sox10), microphthalmia-associated transcription factor (MITF), TRP1, and HMB-45 (Fig. 1D).

### Extracellular environment of limbal melanocytes in situ

**Figure 2:**
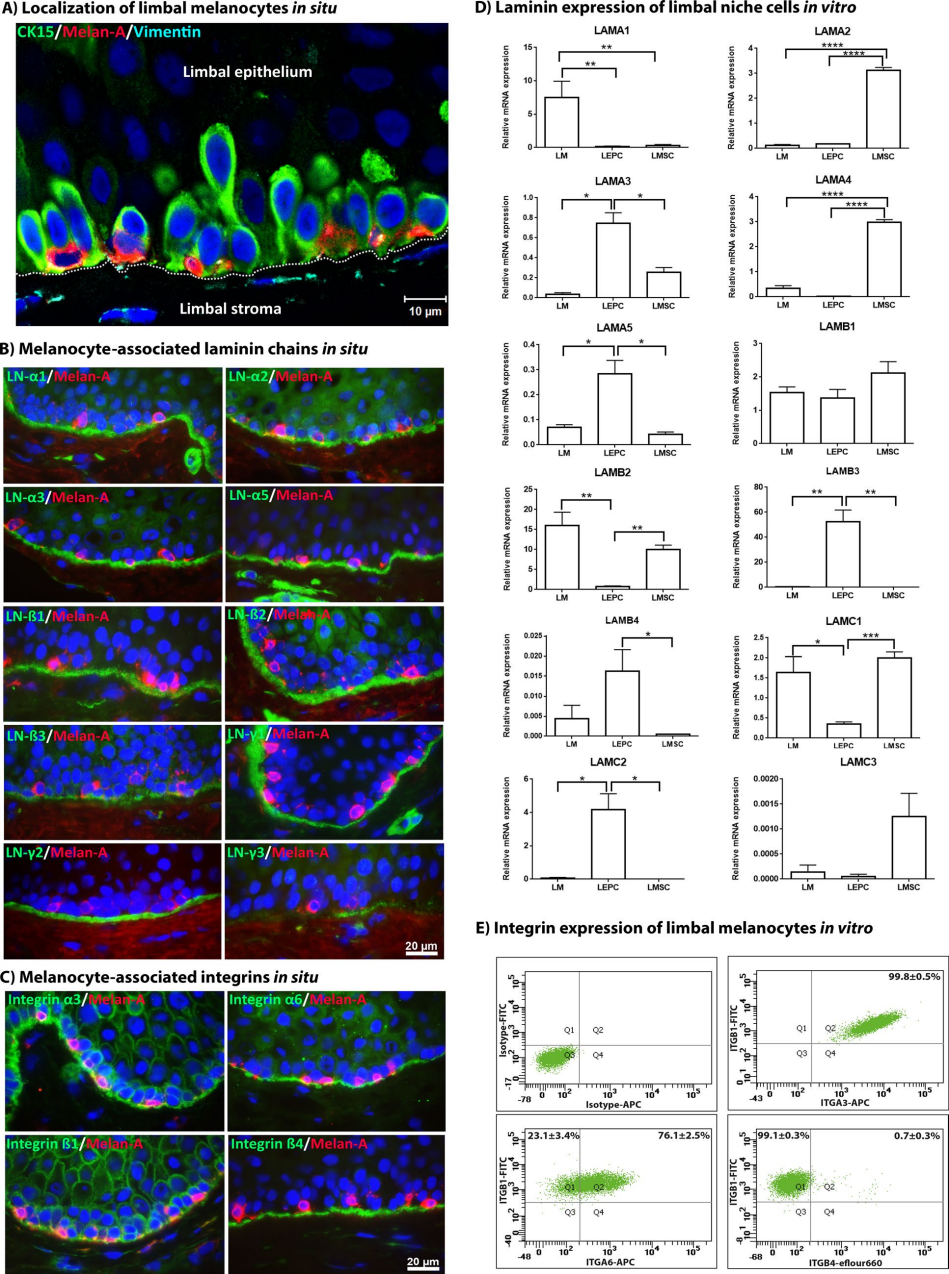
Localization of melanocytes in the limbal niche in situ. (**A**) Immunofluorescence triple staining of corneoscleral tissue sections showing a cell cluster in the basal limbal epithelium containing cytokeratin 15 (CK15)^+^ epithelial stem/progenitor cells (green), Melan-A^+^ melanocytes (red), and vimentin^+^ mesenchymal stromal cells (turquoise); nuclear counterstaining with 4′,6‐diamidino‐2‐phenylindole (DAPI, blue); scale bar = 10 µm; dotted line indicates basement membrane. (**B**) Immunofluorescence double labeling of corneoscleral tissue sections showing staining patterns of laminin (LN)-α1, α2, α3, α5, β1, β2, β3, γ1, γ2 and γ3 in the limbal basement membrane (green) in association with Melan-A^+^ melanocytes (red); nuclei are counterstained with DAPI (blue); scale bar = 20 µm. (**C**) Immunofluorescence double labeling showing staining patterns of integrin α3, α6, β1, and β4 (green) in the basal epithelial cell membranes in association with Melan-A^+^ melanocytes (red); nuclear counterstaining with DAPI (blue); scale bar = 20 µm. (**D)** Quantitative real-time polymerase chain reaction (qRT-PCR) primer assays showing relative expression levels of laminin chains in cultured limbal melanocytes (LM), limbal epithelial progenitor cells (LEPC) and limbal mesenchymal stromal cells (LMSC). Data are normalized to GAPDH and expressed as means (2^−ΔCT^ × 1,000) ± SEM (n = 5). *p < 0.05; **p < 0.01; ***p < 0.001; Mann–Whitney *U* test. (**E**) Flow cytometry analyses of cultured LMs showing expression of integrin α3 (ITGA3), integrin α6 (ITGA6), integrin β1 (ITGB1), and integrin β4 (ITGB4) or isotype control antibodies. Data (% of positive cells) are expressed as means ± SEM (n = 3).

Immunohistochemistry analyses of corneoscleral tissue sections showed that LMs were localized within the basal limbal epithelium in close association with LEPC clusters (Fig. 2A). LMs rested on a basement membrane which contained the LN chains α1, α2, α3, α5, β1, β2, β3, γ1, γ2 and, focally, γ3 (Fig. 2B). They appeared to be anchored to the basement membrane by integrins α3, -α6, and -β1 expressed along their basal cell surface, whereas integrin-ß4 appeared to be not expressed by LMs (Fig. 2C).

Differential gene expression analyses of cultivated LMs in comparison with cultivated LEPCs and LMSCs, derived from the same limbal clusters, showed that LMs predominantly expressed LN-α1 (LAMA1), LN-ß1 (LAMB1), LN-ß2 (LAMB2), and LN-γ1 (LAMC1) (Fig. 2D), suggesting deposition of LN-111 in the limbal basement membrane. By contrast, LEPCs expressed mainly LN-α3, α5, ß1, ß3, ß4, γ1 and γ2, suggesting secretion of LN-332 and LN-511, but potentially also of the rarer isoforms laminin 312 and laminin 512. LMSCs expressed LN-α2, α4, ß1, ß2, γ1 and γ3, indicating contribution of LN-211/221 and LN-411/421 to the basement membrane (Fig. 2D). This differential expression pattern indicates that close interaction between all niche cell types is required to establish the complete molecular composition of the basement membrane in the limbal niche.

As demonstrated by flow cytometry, almost 100% of LMs showed surface expression of integrin α3 and β1, whereas integrin α6 was present on 70–80% of LMs and integrin ß4 was observed on very few cells (0.5–1%) only (Fig. 2E). These findings suggest that attachment of LMs to basement membrane LNs is mediated by integrins a3ß1 and a6ß1.

### Effect of laminin isoforms on melanocyte adhesion, migration, and proliferation in vitro

**Figure 3:**
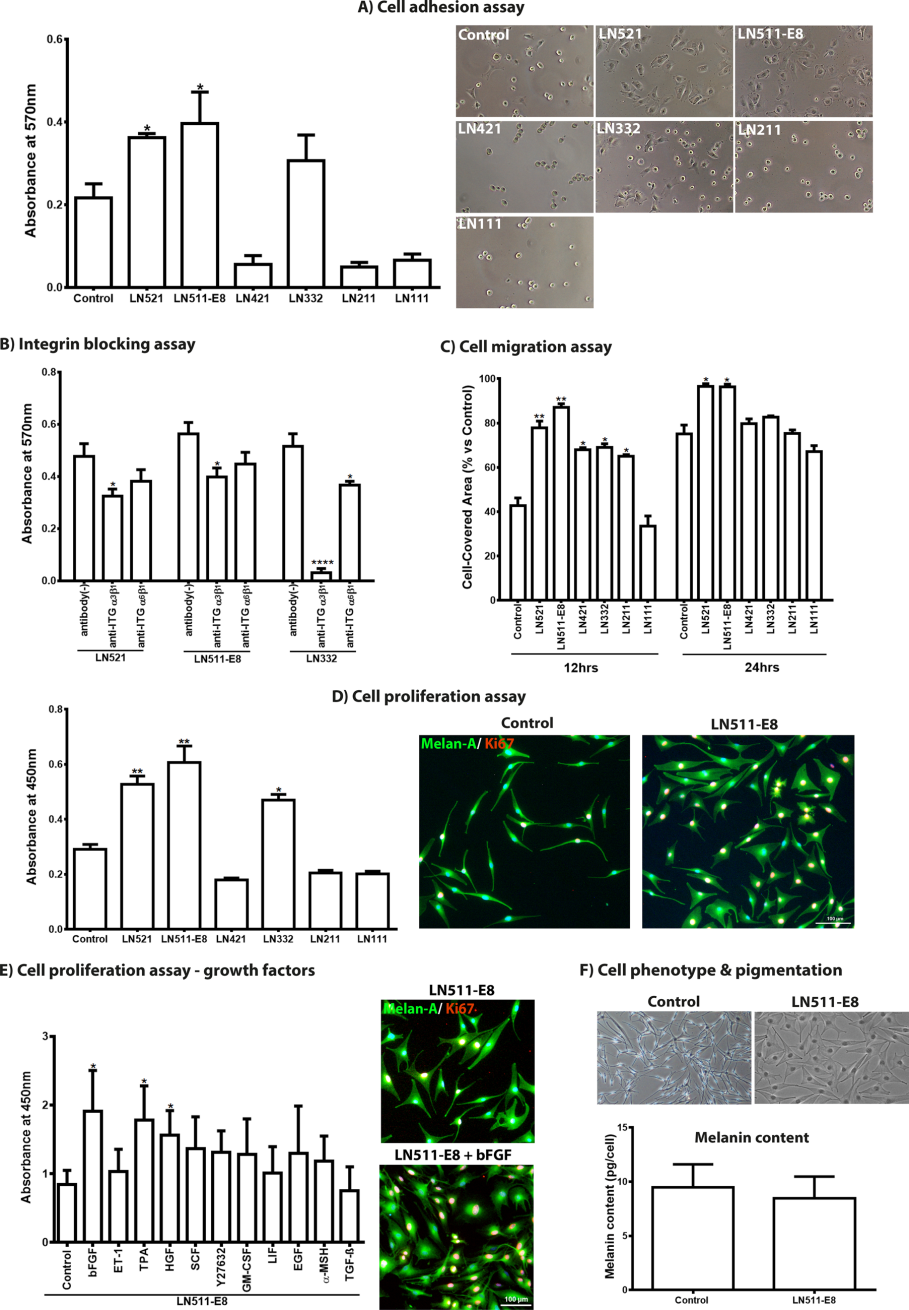
Effect of laminin isoforms on limbal melanocyte function in vitro. (**A**) Effect of laminin (LN) 521, 511-E8, 421, 332, 211, and 111 on cell adhesion compared with control 30 min after seeding. Data are expressed as means ± SEM (n = 4) (left). Phase contrast images of limbal melanocytes after 30 min of adhesion to LN isoforms (right); magnification × 100. (**B**) Functional blocking of integrin-mediated cell adhesion to LN521, LN511-E8, and LN332 was tested using neutralizing antibodies against integrin α3β1 and α6β1 30 min after seeding. Data are expressed as means ± SEM (n = 4). (**C**) The effect of LN isoforms on cell migration was analyzed in two well-culture inserts with a defined cell-free gap and measurement of gap closure 12 and 24 h after removal of the culture inserts. Data are expressed as means ± SEM (n = 3). (**D**) The effect of LN isoforms on cell proliferation was analyzed by BrdU incorporation (left) and immunocytochemical Ki67 expression (right) 72 h after incubation. Data are expressed as means ± SEM (n = 4). Immunocytochemistry shows increased nuclear expression of Ki67 (magenta) in Melan-A^+^ melanocytes (green) grown on LN-511-E8 compared to control; nuclei are counterstained with 4′,6‐diamidino‐2‐phenylindole (DAPI, blue). (**E**) The additional effect of various growth factors on proliferation of melanocytes cultured on LN-511-E8 coated plates was analyzed by BrdU incorporation (left) and immunocytochemical Ki67 expression (right) 48 h after incubation. Data are expressed as means ± SEM (n = 4). Immunocytochemistry shows increased nuclear expression of Ki67 (magenta) in Melan-A^+^ melanocytes (green) cultured in the presence of bFGF; nuclear counterstained with DAPI (blue). (bFGF, basic fibroblast growth factor; EGF, epidermal growth factor; ET-1, endothelin 1; GM-CSF, granulocyte macrophage colony stimulating factor; HGF, hepatocyte growth factor; LIF, leukemia inhibitory factor; α-MSH, α-melanocyte stimulating hormone; SCF, stem cell factor; TGF-ß, transforming growth factor ß1; TPA, 12-*O*-tetradecanoylphorbol 13-acetate; Y-27632, ROCK inhibitor). (**F**) Phase contrast images (top) of melanocytes cultured on LN-511-E8 and tissue culture plastic (control); magnification × 100. Qunatification of cellular melanin content (bottom) in melanocytes cultured on LN-511-E8 and tissue culture plastic (control). Data are expressed as means ± SEM (n = 3). *p < 0.05; **p < 0.01; ****p < 0.001 Mann–Whitney *U* test.

To analyze the effect of LN isoforms on LM function in vitro, we used recombinant human LNs containing α1 (LN-111), α2 (LN-211), α3 (LN-332), and α5 (LN-521, LN-511-E8) chains, since cell binding activities are largely determined by α chains. The effect of the different LN isoforms on cell adhesion was evaluated by determining the number of adherent LMs on LN-coated culture wells at 30 min after seeding compared to uncoated tissue culture plates. Coating with LN-521 and LN-511-E8 increased cell adhesion significantly over uncoated control, whereas LN-332 was slightly better than control and LN-111, LN-211, and LN-421 did not support melanocyte adhesion (Fig. 3A). Phase contrast microscopy showed a more rapid attachment and spreading of LMs on both LN-521 and LN-511-E8 compared to tissue culture plastic. Antibodies against integrin α3β1 showed a significant inhibition of LM binding to LN-332, and to a lesser extent also to LN-521 and LN-511-E8, whereas antibodies against integrin α6β1 only reduced adhesion to LN-332 (Fig. 3B). Thus, LM adhesion to LN-521, LN-511 and LN-332 is mediated mainly through integrin α3β1.

To evaluate the effect of LN isoforms on cell migration, LMs were plated on different LN isoforms and gap closure following removal of a culture insert was analyzed after 12 and 24 h. Except LN-111, all LN isoforms induced a significant increase in cell migration compared to uncoated controls at 12 h, whereas only LN-521 and LN-511-E8 sustained a significant effect on cell migration after 24 h (Fig. 3C). These findings indicate that LN-511, -521 and -332, but not LN-111, support attachment and migration of LMs.

The effect of LN isoforms on cell proliferation was assessed by BrdU incorporation assay 72 h after seeding of LMs on LN-coated culture wells. Compared with uncoated controls, proliferation rates were significantly increased in LMs plated on LN-521 and LN-511-E8 as well as on LN-332 (Fig. 3D). Staining for the proliferation marker Ki-67 confirmed increased numbers of Ki-67 positive nuclei in cells cultured on LN-511-E8 compared to controls (Fig. 3D).

While we cannot definitively define the source of the different laminin isoforms, based on other studies it can be assumed that the LEPCs are a source of the LN-α3 and -α5 containing isoforms^22,23^. In addition, LEPCs produce various soluble factors that may regulate LM functions. Therefore, we assessed an additional effect of soluble factors on cell proliferation by BrdU incorporation assay after seeding of LMs on LN-511-E8-coated culture wells. After 48 h of incubation with various factors known to stimulate proliferation of epidermal melanocytes^24,25^, proliferation rates were significantly increased by basic fibroblast growth factor (bFGF, 2.3-fold) and hepatocyte growth factor (HGF, 1.8-fold) as well as by 12-*O*-Tetradecanoylphorbol-13-acetate (TPA, 2.0-fold) serving as positive control (Fig. 3E)^26^. Although proliferation was also enhanced by stem cell factor (SCF, 1.6-fold), granulocyte–macrophage colony-stimulating factor (GM-CSF, 1.4-fold), epidermal growth factor (EGF, 1.3-fold), α-melanocyte stimulating hormone (α-MSH, 1.3-fold) and the Rock inhibitor Y27632 (1.4-fold), differences were not statistically significant (Fig. 3E). Staining for the proliferation marker Ki-67 confirmed increased numbers of Ki-67 positive nuclei in cells cultured on LN-511-E8 in presence of bFGF compared to LN-511-E8 alone (Fig. 3E).

Compared to uncoated tissue culture plastic, LN-511-E8 also supported the formation of a multidendritic flattened LM phenotype, indicating enhanced substrate adhesion, without affecting melanin synthesis as reflected by the melanin content per cell (LN-511-E8: 9.4 ± 2.2 pg/cell; control: 8.4 ± 2.1 pg/cell) (Fig. 3F).

### Utilization of limbal melanocytes for corneal epithelial tissue engineering

**Figure 4:**
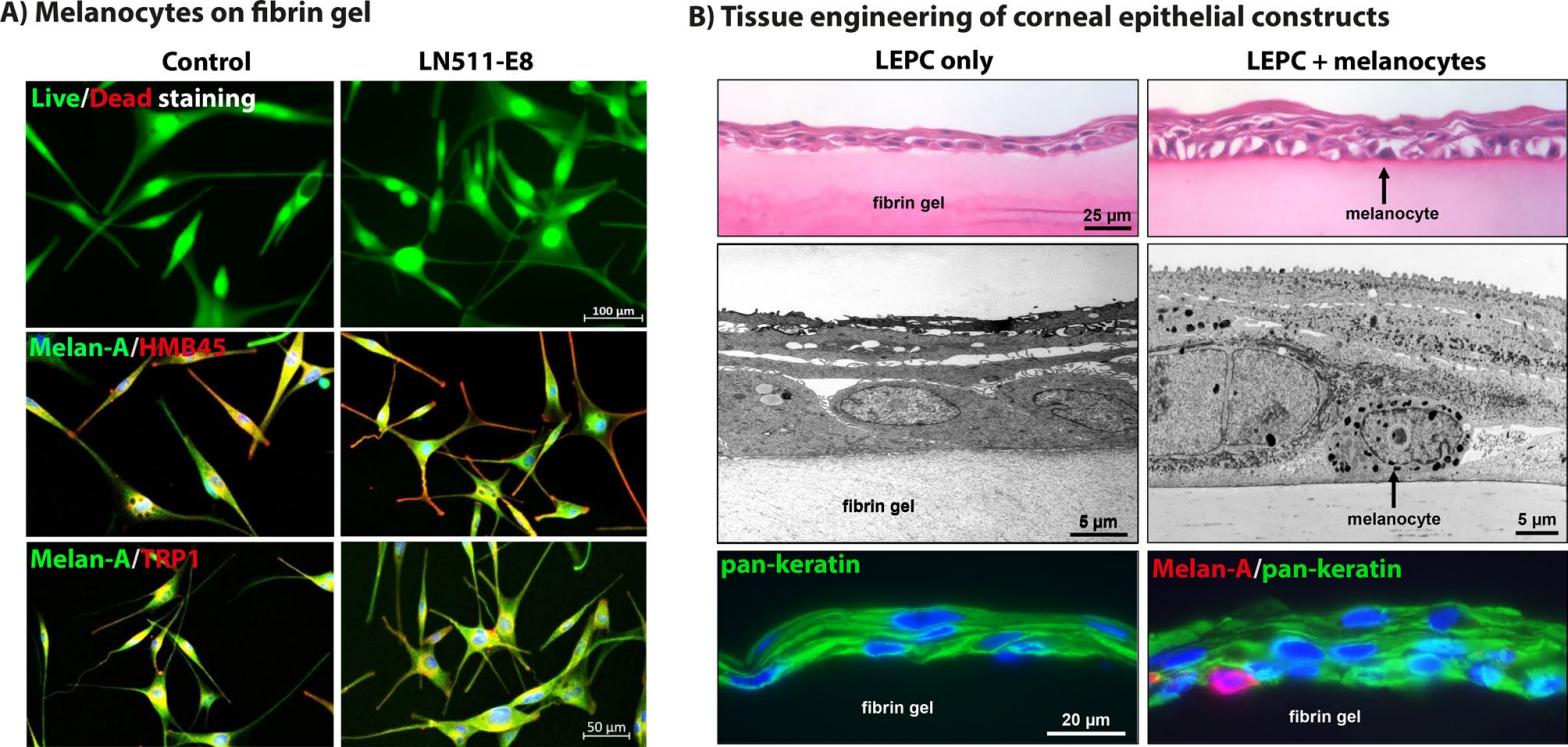
Corneal epithelial tissue engineering using limbal melanocytes and LN-511-E8. (**A**) Live/dead viability assay and immunofluorescence double labeling studies after 24 h of cultivation of limbal melanocytes on fibrin hydrogels pretreated with LN511-E8 or untreated fibrin gels (control). In both conditions, cells are viable and co-express melanocyte markers Melan-A (green) in association with HMB-45 and tyrosinase related protein 1 (TRP1) (red), respectively; nuclear counterstaining with 4′,6‐diamidino‐2‐phenylindole (DAPI, blue). (**B**) Light microscopic analysis of fibrin-based epithelial constructs formed by limbal epithelial progenitor cells (LEPC) without or with co-cultured melanocytes after 10 days of cultivation (top). Transmission electron microscopy images of epithelial constructs without or with basally located melanocytes (center). Immunofluorescence analysis of epithelial constructs showing epithelial cells positive for the epithelial marker pan-keratin (green) in association with Melan-A-positive melanocytes (red) localized within the basal cell layer (bottom); nuclear counterstain with DAPI (blue).

To generate corneal epithelial constructs suitable for clinical application, LEPCs were co-cultivated with or without mitotically active LMs pre-seeded on fibrin hydrogels that were pre-incubated with LN-511-E8. LMs pre-seeded on LN-511-E8 containing fibrin gels acquired a polydendritic well-adherent phenotype compared to a mostly bipolar phenotype on untreated control gels (Fig. 4A). However, cell viability and expression of melanocyte markers, such as Melan-A, HMB-45 and TRP1, were not different between LN-511-E8 containing and untreated control gels (Fig. 4A).

Light microscopic analyses of tissue-engineered epithelial constructs showed a multilayered cell sheet consisting of a cuboidal basal layer and 4 to 6 layers of flattened suprabasal cells upon co-culture with LMs, but only 3 to 4 cell layers without LMs, after 8–10 days of cultivation (Fig. 4B, top). Transmission electron microscopy confirmed formation of well-organized stratified epithelial cell sheets with LMs residing within the basal layer in intimate contact with the LN-511-E8 coated gel surface (Fig. 4B, center). Immunofluorescence analysis of epithelial constructs showed epithelial cells expressing the epithelial marker pan-keratin in association with Melan-A positive LMs localized within the basal cell layer (Fig. 4B, bottom).

These data suggest that co-cultivation of LEPCs with LMs on LN-511-E8-containing fibrin gels promote the generation of a multilayered stratified corneal epithelial tissue equivalent, which partly mimics the native niche and which may be suitable for transplantation and ocular surface reconstruction.

## Discussion

Stem cell-based tissue engineering aims to mimic the native stem cell niche and to present the appropriate microenvironmental cues, including supporting niche cell populations and matrix components, in order to maintain stem cell function within the graft^27^. We have previously reported that fibrin-based hydrogels incorporating the limbus-specific LN-511 isoform and its biologically active C-terminal domain (E8 fragment) resulted in multilayered stratified corneal epithelial constructs after 14 days in culture^18^. An additional incorporation of supporting niche cells along with their secretome into such prefunctionalized hydrogels would be a further significant step towards an organotypic culture system. Whereas the beneficial effect of the LMSC niche cell population on LEPC expansion has been well documented^12–14,28–30^, LMs have been only rarely used for LEPC co-cultivation^9,10^, although their intimate spatial association with LEPCs in vivo anticipates important biological roles besides photoprotection^8,10^.

In order to obtain sufficient numbers of pure LM populations for both functional studies and tissue engineering approaches, we established a method for the efficient cultivation of primary human melanocytes from the corneoscleral limbus. We used collagenase to enzymatically isolate limbal cell clusters from limbal tissue specimens as initially described by Chen et al.^31^ and expanded the individual stem and niche cell populations using cell type-specific media and purification steps. Contaminating epithelial cells were readily removed from the melanocyte cultures by low concentrations of trypsin, which preferentially detaches melanocytes and stromal cells^26^. The major obstacle to obtain pure cultures of melanocytes is, however, contamination with fibroblast-like stromal cells, which grow faster than melanocytes. Therefore, treatment with low concentrations of geneticin has been shown to effectively inhibit the proliferation of fibroblasts within mixed cultures without harming melanocytes^10,32–34^. In our experience, two to three cycles of geneticin are required to remove contaminating fibroblasts completely. The highly enriched LM populations expressed all known melanocyte markers, i.e., Melan-A, CD117 (c-Kit), TRP1, MITF, Sox10, and HMB-45 by flow cytometry, RT-PCR and immunocytochemistry^21^. Pure LM populations were maintained in melanocyte-specific CnT-40 medium containing 1% serum and growth factors including ET-1/3 for up to 24 months. These stable, long-lived cell cultures can be durably used for functional studies or tissue engineering approaches, although they exhibit signs of growth arrest by contact inhibition. Therefore, we never observed any increased cell proliferation in long term melanocyte cultures indicative of potential malignant progression. Moreover, we did not observe any features of nuclear atypia, such as prominent nucleoli, binucleation or nuclear budding, in cultured melanocytes by phase-contrast microscopy or DAPI staining. Hence we ruled out any possible concerns of genomic instability and malignant progression in long term cultured melanocytes.

Many attempts have been made to stimulate melanocyte proliferation in vitro, mostly by addition of mitogens, such as TPA, bFGF, ET-1, HGF, SCF and α-MSH^24,25^. We confirmed a stimulatory effect of bFGF, HGF, TPA and SCF on LM proliferation, but we consider these soluble factors rather unsuitable for tissue engineering strategies, because of the need to replenish growth factors and difficulties in standardizing growth factor concentrations. In contrast, in vitro reconstitution of the extracellular matrix with its intrinsic regulatory functions on niche cell populations has received increasing attention for tissue engineering applications^35^. Melanocytes, both at the limbus and in the epidermis, adhere to epithelial basement membranes with LN binding integrins such as α3β1 and α6β1^36,37^. We hypothesized that LN isoforms enriched in the limbal niche not only regulate LEPC but also LM functions through interaction with integrin receptors. Notably, LMs displayed highest adhesion, migration and proliferation rates on LN-α5 and LN-α3 containing substrates, i.e. LN-521, LN-511-E8 and LN-332, which are likely to be produced by LEPCs, but not on LN-111, LN-211 or LN-411, which we assume to be produced by LMs or LMSCs, respectively. These observations are consistent with reports on epidermal melanocytes, showing that keratinocyte-derived LN-511 and LN-332 regulate melanocyte adhesion, migration, differentiation and melanin production in the skin^15–17^. In contrast to epithelial cells or fibroblasts, which express integrins to adhere to their own secreted extracellular matrix, epidermal and limbal melanocytes mainly respond to matrix components previously deposited by epithelial cells^38^. The particular characteristics of LN-511 and its C-terminal integrin-binding domain (E8 fragment), which are recognized by α3β1 and α6β1 integrins expressed on LEPC and LMs, and which have been qualified to optimally support LEPC expansion in vitro, render these matrix proteins optimally suited to co-cultivation approaches^18,39^. Particularly the recombinant LN-511-E8 fragment is promising tool for stem cell-based tissue engineering^40^.

Using pure LM and LEPC populations derived from the same limbal cell clusters, we showed that LEPCs co-cultivated with LMs on LN-511-E8 pre-incubated fibrin scaffolds showed superior growth capacity and stratification over LEPC monocultures after 8–10 days of cultivation. Melanocytes could be detected in the basal layer of the epithelial constructs resting on the LN-511-E8 coated gel surface, thereby closely resembling the in vivo situation. Although not yet tested, the presence of melanocytes may significantly enhance the durability of the tissue equivalents, because epithelial cell sheets that contained LEPC and melanocytes coincidentally could be maintained in culture for more than 1 year^41^. The results suggest that epithelial stem/progenitor cells and melanocytes may act in concert both in the native limbal niche and in tissue engineered epithelial sheets. Human melanocytes have been also integrated into tissue engineered epidermal and skin equivalents, which have been successfully used to repair skin defects^42–44^. In these in vitro models, melanocytes have been shown to function in a similar manner to that in vivo.

In conclusion, we generated limbal epithelial constructs by co-cultivation of pure populations of human LMs and LEPCs on LN-511-E8 coated fibrin scaffolds. By reproducing physiological cell–cell and cell–matrix interactions of the native niche environment, these biomimetic co-culture systems provide a promising experimental model for investigating the functional role of melanocytes in the limbal stem cell niche, the pathogenesis of melanocytic tumors originating at the limbus^45^, and their suitability for developing advanced therapy medical products. Future translation of these constructs into clinical application is expected to improve long-term outcomes of limbal stem cell transplantation for ocular surface reconstruction.

## Methods

### Human tissues and study approval

Human donor corneas with appropriate research consent were procured by the Erlangen Cornea Bank after corneal endothelial transplantation. Tissues were obtained after informed written consent from the relatives of the donors and used in accordance with the principles of the Declaration of Helsinki for experiments involving human tissues and samples. Ethics approval was obtained from the Institutional Review Board of the Medical Faculty of the University of Erlangen-Nürnberg (No. 4218-CH).

### Cell culture

Limbal tissue specimens were prepared as previously described^18^. Briefly, corneoscleral buttons were rinsed in Hanks' balanced salt solution and cut into 12 one-clock-hour sectors, from which limbal segments were obtained by incisions made at 1 mm before and beyond the anatomical limbus. Each limbal segment was enzymatically digested with 2 mg/ml collagenase A (Roche Diagnostics, Mannheim, Germany) at 37 °C for 18 h to generate LEPC-LMSC-LM containing cell clusters. Cell clusters were isolated by using reversible cell strainers with a pore size of 37 µm (Stem Cell Technologies, Köln, Germany) and further dissociated into single cells by digestion with 0.25% trypsin and 0.02% EDTA (Pan Biotech, Aidenbach, Germany) at 37 °C for 10–15 min. Single cell suspensions were seeded into T75 flasks (Corning, Tewksbury, MA) and either grown in keratinocyte serum free medium (KSFM) supplemented with bovine pituitary extract, epidermal growth factor (Life Technologies, Carlsbad, CA) and 1 × penicillin–streptomycin–amphotericin B mix (Pan Biotech, Aidenbach, Germany) to enrich the LEPC population, in Mesencult medium (Stem Cell Technologies, Köln, Germany) to enrich the LMSC population, or in CnT-40 medium containing 1% serum and endothelin-1 and -3 (CellnTech, Bern, Switzerland) to enrich the LM population. Flasks were incubated at 37 °C under 5% CO_2_ and 95% humidity, and media were changed every second day.

After 10–12 days, melanocyte-like cells and stromal fibroblasts were enzymatically separated from contaminating epithelial cells by using a solution of 0.05% trypsin-0.01% EDTA (Life Technologies) and re-seeded into a T75 flask in CnT-40 medium. After reaching 80% of confluency, mixed cell cultures were treated with 0.2 mg/ml geneticin (Life Technologies), an inhibitor of protein synthesis, in medium 254 (Life Technologies) for 48 h to remove contaminating stromal fibroblasts. Geneticin treatments were repeated up to three times until pure LM cultures were obtained. At this concentration, geneticin has a very limited toxicity for melanocytes showing lower protein synthesis, but causes harm to actively synthesizing fibroblasts and stromal cells^30,31^.

### Cell adhesion assay

Cell adhesion assays were performed as described previously^18^. Briefly, LMs isolated from corneoscleral buttons as described above were seeded onto 96 well-plates coated with 1.0 µg/cm^2^ human recombinant LN-111, -211, -332, -421, -521 (BioLamina, Sundbyberg, Sweden), and 0.5 µg/cm^2^ recombinant LN-511-E8 (Nippi, Tokyo, Japan) as per manufacturers’ recommendations. Cells were seeded at a density of 50,000 cells/cm^2^ and left to adhere for 30 min at 37 °C. Standard tissue culture treated plates were used as control. After incubation, plates were washed with Dulbecco's Phosphate-Buffered Saline (DPBS) using a Static Cell Adhesion Wash Chamber (Glycotec, Maryland, USA) to remove non-adherent cells. Adherent cells were fixed with 4% paraformaldehyde/DPBS for 15 min and stained with 0.1% crystal violet for 20 min. After three washes with water, stained cells were extracted with 1% sodium dodecyl sulfate and quantified by measuring optical density (OD) at 570 nm using a spectrophotometer (Multiskan Spectrum; Thermo Scientific, Waltham, USA). All experiments were performed in quadruplicates. The fold change values were calculated as OD of the LN/OD of control.

The effect of an interaction between cellular integrins and extracellular LNs on cell adhesion was evaluated by seeding LMs (50,000 cells/cm^2^) in 96-well plates coated with recombinant LN-332, -521, and LN-511-E8 in the presence or absence of integrin-neutralizing antibodies, i.e., anti-α3 integrin (20 µg/ml) (Merck Millipore, Darmstadt, Germany), anti-α6 integrin (20 µg/ml) (Merck Millipore) and anti-β1 integrin (2.5 µg/ml) (R&D Systems Inc., Minneapolis, MN, USA). 60 min after seeding, the numbers of adherent cells were determined as described above.

### Cell migration assay

To exactly measure the change in the cell-covered area over time, 2 well-culture inserts with a defined cell-free gap were used (ibidi GmbH, Planegg, Germany) and assays performed as described previously^18^. Briefly, the wells were coated with LN isoforms as described above and seeded with 70 µl of a LM suspension containing 5 × 10^5^ cells/ml. After formation of a cellular monolayer (24 h), the silicone inserts were removed and the culture medium was supplemented with 2.5 µg/ml of soluble LNs. Images of each well were acquired immediately following insert removal (0 h) and after 12 and 24 h by using an inverted microscope (BX51; Olympus, Hamburg, Germany). Image analysis software Cell^˄^F (Olympus) was used to measure areas that were free of migrating cells. All experiments were performed in triplicates.

### Cell proliferation assay

The effect of LNs on LM proliferation was quantified using the Cell Proliferation ELISA BrdU Colorimetric Assay Kit (Roche Diagnostics, Mannheim, Germany) as previously described^18^. Cells were seeded into 96-well plates pre-coated with LN isoforms at a density of 5,000 cells/well, cultured for 72 h, and labeled with BrdU according to the manufacturer’s instructions. Absorbance was measured at 450 nm using a spectrophotometer (Multiskan Spectrum; Thermo Scientific, Waltham, MA), and fold change values were calculated as described above. Experiments were performed in quadruplicates.

To test any additional effect of growth factors on cell proliferation, LMs were cultivated on LN-511-E8 coated 96 well plates. After 24 h of plating, cells were incubated with α-melanocyte stimulating hormone (α-MSH; 166 ng/ml), 12-*O*-tetradecanoylphorbol 13-acetate (TPA; 10 ng/ml), endothelin 1 (ET-1; 25 ng/ml), granulocyte macrophage colony stimulating factor (GM-CSF; 5 ng/ml), leukemia inhibitory factor (LIF; 5 ng/ml), epidermal growth factor (EGF; 5 ng/ml), hepatocyte growth factor (HGF; 5 ng/ml), stem cell factor (SCF; 5 ng/ml), basic fibroblast growth factor (bFGF; 3 ng/ml), transforming growth factor beta 1 (TGF-ß1; 5 ng/ml) or ROCK inhibitor (Y-27632; 10 µM) for 48 h and labeled with BrdU according to the manufacturer’s instructions. Absorbance was measured at 450 nm using a spectrophotometer (Multiskan Spectrum), and fold change values were calculated as described above. Experiments were performed in quadruplicates.

For immunocytochemical analysis of cell proliferation, LMs were seeded at a density of 10,000 cells/well into 4 well-chamber slides (LabTek; Nunc, Wiesbaden, Germany), cultured for 72 h, stained with anti-Ki-67 antibody (Abcam; Cambridge, UK), and counted using Cell^F image analysis software (Olympus).

### Pigmentation assay

The effect of LNs on melanin production was determined using a modified protocol described by Friedmann and Gilchrest^46^. Briefly, cultured cells were washed with DPBS, incubated with Trypsin–EDTA, and pelleted by centrifugation at 500 g for 10 min. Supernatants were discarded, and cell pellets were washed with DPBS before being dissolved in 1 ml of 1 N NaOH/10% dimethyl sulfoxide (DMSO) by shaking vigorously for 2 h at 80 °C. Following incubation, the samples were centrifuged at 12,000*g* for 10 min, and supernatants were transferred to 96 well plates. Melanin concentration of samples was determined by comparing absorbance at 470 nm (Multiskan Spectrum) with a standard curve generated from synthetic melanin (Merck, Darmstadt, Germany).

### Flow cytometry

LMs were characterized by flow cytometry using fluorochrome labelled antibodies and isotype control antibodies (BD Biosciences, Heidelberg, Germany) as previously described^18^. Single cell suspensions (0.5–1 × 10^6^ cells) were incubated with saturating concentrations of conjugated antibodies in 100 µl DPBS, 0.1% sodium azide and 2% fetal calf serum for 20 min. After three washes, the cells were centrifuged at 200×*g* for 5 min. Cells were re-suspended in ice-cold DPBS containing 5 µl of 7-amino-actinomycin D (7-AAD) to exclude dead cells. Cytometry was performed on a FACSCanto II (BD Biosciences) by using FACS Diva Software. A total of 10,000 events were acquired to determine the positivity of cell surface markers.

### Real time RT-PCR

RNA isolation from cultured LMs was performed using the RNeasy Micro Kit (Qiagen, Hilden, Germany) including an on-column DNase digestion step according to the manufacturer’s instructions. First-strand cDNA synthesis was performed using 5 µg of RNA from cultured cells and Superscript II reverse transcriptase (Invitrogen, Karlsruhe, Germany) as previously described^18^. PCR reactions were run in triplicate in 1 × TaqMan Probe Mastermix (Roche Diagnostics), according to the manufacturers’ recommendations. Primer sequences (Eurofins, Anzing, Germany) are given in Table 1. For normalization of gene expression levels, ratios relative to the housekeeping gene *GAPDH* were calculated by the comparative *C*_T_ method (ΔΔ*C*_T_). Genes were considered as differentially expressed when their expression levels exceeded a two-fold difference in all specimens analyzed.

**Table 1:** Primers used in qRT-PCR primer assays.

Gene symbol	Accession no	Product length	Probe no./SYBR	Sequence 5′–3′
GAPDH	NM_002046.3	72	P3	CAGCAAGAGCACAAGAGGAA
				GTGGTGGGGGACTGAGTGT
KIT	NM_000222.2	61	P6	GAGTAGCTTACCAGAAGCTTCCATAG
				CATAGGGACTGATGCCTTCC
KRT3	NM_057088.2	92	P74	CCTGTGATTGTCCAGGTGTG
				ATACATCAGAGCTGTAGTGAGCATC
KRT15	NM_002275.3	60	P10	CATTGGCATCAGGGAAGC
				TTGATGTGGAAATTGCTGCT
LAMA1	NM_005559.3	60	P38	AGGATGACCTCCATTCTGACTT
				CCTTACATGGGCACTGACCT
LAMA2	NM_000426.3	68	P81	GCAAGCCACTGGAGGTTAAT
				TGGGCATGATACAGGTTGAA
LAMA3	NM_198129.1	65	P29	CCAGGAATATGGGTTGCTTG
				GGGAGCAGCACCAGGTAAT
LAMA4	NM_001105209.1	68	P55	GCCACACTCGTCCTTCTCTC
				CCCAGGTGAAACTCTCAAGG
LAMA5	NM_005560.3	75	P44	ATGACTCGCTCTGTGGAGGT
				GGGGTTGGCTGTGTCCTA
LAMB1	NM_002291.2	72	P82	AAGCCAGAAAGTTGCTGTGTATAG
				CCTTGTTCACCTCAGCCATT
LAMB2	NM_002292.3	100	P43	AAGGCCTACCCCAGTTCCTA
				GGGTTCACACTGGTTTATTGG
LAMB3	NM_001127641.1	72	P23	GGCATGCCATTGAAACTAAGA
				AGAACTAAAGGCGGGGGATA
LAMB4	NM_007356.2	67	P5	CCCCACACCCTGTCCTTATT
				ATTTTCCTGGTGGCATTTCA
LAMC1	NM_002293.3	92	P66	GCCATTATTTTATTGTCTAGCTCCA
				ATCCCTGTGTCAACCAGCAT
LAMC2	NM_005562.2	96	P65	CACTCTGTGCCTTTCTACAACTG
				CCAAGGTGGAAGTGCCTCT
LAMC3	NM_006059.3	67	P14	CAGGACTCCTCAGCATTTCC
				TTGCCATCTGCTGGAAGAG
MLANA	NM_005511.1	79	P39	GAGAAAAACTGTGAACCTGTGGT
				AAGGTGGTGGTGACTGTTCTG
NT5E	NM_002526.4	111	P80	CCAGTCCACTGGAGAGTTCC
				CGACACTTGGTGCAAAGAAC
TYRP1	NM_000550.2	77	P2	CCTGTGACCAGAGGGTTCTC
				CCGGACAAAGTGGTTCTTTTC

Primers were used for Probe based (Universal Probe Library) or SYBR Green based qRT-PCR assays with an annealing temperature of 60 °C.

### Tissue engineering of fibrin-based epithelial constructs

Scaffolds for tissue engineering and 3D-cell culture were prepared from fibrin as previously described^18^. Briefly, fibrin hydrogels were prepared by dissolving fibrinogen and thrombin stock solutions (Tisseel; Baxter Deutschland GmbH, Unterschleißheim, Germany) in 1.1% NaCl and 1 mM CaCl_2_ to a final concentration of 10 mg/ml fibrinogen and 3 IU/ml thrombin. Recombinant LN-511-E8 (10 µg/ml) was incorporated into the gels, which were placed into 24 well-culture inserts and allowed to polymerize at 37 °C. After washing with DPBS, gels were additionally coated with LN-511-E8 (5 µg/ml) overnight. LN-511-E8 free gels served as controls. LMs were seeded onto LN-coated and uncoated control gels at a density of 5 × 10^4^ cells/cm^2^ and cultivated in CnT-40 medium for 24 h. Then, LEPCs were seeded at a density of 1 × 10^5^ cells/cm^2^ on top of fibrin gels with and without LMs and were cultivated in DMEM/Ham’s F12 (Hyclone; GE Health Care Life Sciences, Freiburg, Germany) supplemented with Human Corneal Growth Supplement (Life Technologies). After 48 h, the fibrin constructs were raised to the air–liquid interface and cultured for further 6 to 8 days. Finally, fibrin gels were fixed for light and electron microscopy and immunohistochemistry as described below.

### Histology

For light and electron microscopic analyses, fibrin gels were fixed in 2.5% glutaraldehyde in 0.1 M phosphate buffer, dehydrated, and embedded in paraffin or epoxy resin, respectively, according to standard protocols^18^. Paraffin sections were stained with hematoxylin and eosin, and ultrathin resin sections were stained with uranyl acetate-lead citrate and examined with an electron microscope (EM 906E; Carl Zeiss Microscopy, Oberkochen, Germany).

### Immunohisto- and immunocytochemistry

Corneoscleral tissue samples obtained from 10 normal human donor eyes (mean age, 75.6 ± 10.3 years; fixed within 15 h post-mortem) and fibrin-based 3D-cultures were embedded in optimal cutting temperature (OCT) compound and frozen in isopentane-cooled liquid nitrogen. As previously described^18^, cryosections of 4 μm thickness were fixed in cold acetone for 10 min, blocked with 10% normal goat serum, and incubated in primary antibodies (Table 2) diluted in DPBS overnight at 4 °C. Antibody binding was detected by Alexa 488-conjugated secondary antibodies (Molecular Probes, Eugene, OR) and nuclear counterstaining was performed with DAPI (Sigma-Aldrich, St. Louis, Missouri). Immunolabelled cryosections and cultured LMs were examined with a fluorescence microscope (Olympus BX51) or a laser scanning confocal microscope (LSM 780; Carl Zeiss Microscopy). In negative control experiments, the primary antibodies were replaced by equimolar concentrations of an irrelevant isotypic primary antibody.

**Table 2:** List of antibodies used.

Antibody (clone), Host species	Antibody concentration	Application	Antibody source
CD49c (Integrin α3) APC (P1B5), mouse	1.25 µg/ml	Flow cytometry	eBioscience
CD49f (Integrin α6) APC (GoH3), rat	1.25 µg/ml	Flow cytometry	eBioscience
CD29 (Integrin β1) FITC (TS2/16), mouse	2.5 µg/ml	Flow Cytometry	eBioscience
CD104 (Integrin β4) eFluor 660 (439-9B), rat	10 µg/ml	Flow cytometry	eBioscience
CD54 (ICAM-1) APC (HA58), mouse	10 µg/ml	Flow cytometry	BD
CD117 (c-Kit) APC (YB5.B8), mouse	10 µg/ml	Flow cytometry	BD
IgG1 isotype PE (MOPC-21), mouse	5 µg/ml	Flow cytometry	BD
IgG1 isotype FITC (IS5-21F5), mouse	2.5 µg/ml	Flow cytometry	Miltenyi Biotec
IgG1 isotype APC (IS5-21F5), mouse	1.25–2.5 µg/ml	Flow cytometry	Miltenyi Biotec
IgG2b isotype APC (141945), rat	1.25–10 µg/ml	Flow cytometry	R&D systems
Melan-A PE (M2-7C10 + M2-9E3), mouse	10 µg/ml	Flow cytometry	Novus biologicals
Integrin α3 (ASC-1), mouse	20 µg/ml	Blocking	Millipore
Integrin α6 (NKI-GoH3), rat	20 µg/ml	Blocking	Millipore
Integrin β1 (P5D2), mouse	2.5 µg/ml	Blocking	R&D systems
CD117/c-Kit (Ab81), mouse	1:500	Immunohisto/cytochemistry	Cell signaling
Cytokeratin pan (AE1/AE3), mouse	1:100	Immunohistochemistry	Dako
Cytokeratin 15 (LHK15), mouse	1:500	Immunohisto/cytochemistry	Abcam
HMB-45 (HMB45), mouse	1:50	Immunohisto/cytochemistry	Dako
Integrin α3 (P1B5), mouse	1:200	Immunohistochemistry	Dako
Integrin α6 (GoH3), rat	1:100	Immunohistochemistry	Chemicon/Millipore
Integrin β1 (HB1.1), mouse	1:500	Immunohistochemistry	Chemicon/Millipore
Integrin β4 (439-9B), rat	1:200	Immunohistochemistry	Chemicon/Millipore
Ki-67 (SP6), rabbit	1:1,000	Immunocytochemistry	Abcam
Laminin α1 (317), rabbit	1:500	Immunohistochemistry	L. Sorokin
Laminin α2 (401), rabbit	1:500	Immunohistochemistry	L. Sorokin
Laminin α3, rabbit	1:6,000	Immunohistochemistry	R. Timpl/T. Sasaki
Laminin α5 (405), rabbit	1:4,000	Immunohistochemistry	L. Sorokin
Laminin β1 (IIID9), mouse	Undiluted	Immunohistochemistry	L. Sorokin
Laminin β2 (409), rabbit	1:200	Immunohistochemistry	L. Sorokin
Laminin β3, rabbit	1:6,000	Immunohistochemistry	R. Timpl/T. Sasaki
Laminin γ1 (3E10), rat	undiluted	Immunohistochemistry	L. Sorokin
Laminin γ2 (LE4-6), rabbit	1:4,000	Immunohistochemistry	R. Timpl/T. Sasaki
Laminin γ3, rabbit	1:2000	Immunohistochemistry	R. Timpl/T. Sasaki
Melan-A (A103), mouse	1:25	Immunohisto/cytochemistry	Dako
Melan A, (EP1422Y), rabbit	1:500	Immunohisto/cytochemistry	Abcam
MITF (C5), mouse	1:500	Immunohisto/cytochemistry	Abcam
Nestin (10C2), mouse	1:100	Immunohisto/cytochemistry	Abcam
Sox10 (BC34), mouse	1:50	Immunohisto/cytochemistry	Abcam
TRP1 (TA99), mouse	1:100	Immunohisto/cytochemistry	Abcam
Vimentin, (280618), rat	1:50	Immunohisto/cytochemistry	R&D Systems

### Statistical analysis

Statistical analyses were performed using the GraphPad InStat statistical package for Windows (Version 8.3.0; GraphPad Software Inc., La Jolla, CA). Data are expressed as mean ± standard error of the mean from individual experiments. Group comparisons were performed using an unpaired two-tailed *t* test or a Mann–Whitney *U* test. A *p* value of < 0.05 was considered statistically significant.
